# Phencyclidine Disrupts Neural Coordination and Cognitive Control by Dysregulating Translation

**DOI:** 10.1016/j.bpsgos.2023.04.009

**Published:** 2023-05-31

**Authors:** Eun Hye Park, Hsin-Yi Kao, Hussam Jourdi, Milenna T. van Dijk, Simón Carrillo-Segura, Kayla W. Tunnell, Jeffrey Gutierrez, Emma J. Wallace, Matthew Troy-Regier, Basma Radwan, Edith Lesburguères, Juan Marcos Alarcon, André A. Fenton

**Affiliations:** a Center for Neural Science, New York University, New York, New York; b Graduate Program in Neuroscience and Physiology, New York University Langone Medical Center, New York, New York; c Graduate Program in Mechanical and Aerospace Engineering, New York University Tandon School of Engineering, New York, New York; d Graduate Program in Neural and Behavioral Science, State University of New York, Downstate Health Sciences University, Brooklyn, New York; e Department of Physiology and Pharmacology, State University of New York, Downstate Health Sciences University, Brooklyn, New York; f Graduate Program in Neural Science, Center for Neural Science, New York University, New York, New York; g Department of Pathology, State University of New York, Downstate Health Sciences University, Brooklyn, New York; h Robert F. Furchgott Center for Neural and Behavioral Science, State University of New York, Downstate Health Sciences University, Brooklyn, New York; i Neuroscience Institute, NYU Langone Health, New York, New York

**Keywords:** Cognitive coordination, mGluR1/5, Neural discoordination, NR2A, Protein synthesis, Translation machinery

## Abstract

**Background:**

Phencyclidine (PCP) causes psychosis, is abused with increasing frequency, and was extensively used in antipsychotic drug discovery. PCP discoordinates hippocampal ensemble action potential discharge and impairs cognitive control in rats, but how this uncompetitive NMDA receptor (NMDAR) antagonist impairs cognition remains unknown.

**Methods:**

The effects of PCP were investigated on hippocampal CA1 ensemble action potential discharge in vivo in urethane-anesthetized rats and during awake behavior in mice, on synaptic responses in ex vivo mouse hippocampus slices, in mice on a hippocampus-dependent active place avoidance task that requires cognitive control, and on activating the molecular machinery of translation in acute hippocampus slices. Mechanistic causality was assessed by comparing the PCP effects with the effects of inhibitors of protein synthesis, group I metabotropic glutamate receptors (mGluR1/5), and subunit-selective NMDARs.

**Results:**

Consistent with ionotropic actions, PCP discoordinated CA1 ensemble action potential discharge. PCP caused hyperactivity and impaired active place avoidance, despite the rodents having learned the task before PCP administration. Consistent with metabotropic actions, PCP exaggerated protein synthesis–dependent DHPG-induced mGluR1/5-stimulated long-term synaptic depression. Pretreatment with anisomycin or the mGluR1/5 antagonist MPEP, both of which repress translation, prevented PCP-induced discoordination and the cognitive and sensorimotor impairments. PCP as well as the NR2A-containing NMDAR antagonist NVP-AAM077 unbalanced translation that engages the Akt, mTOR (mechanistic target of rapamycin), and 4EBP1 translation machinery and increased protein synthesis, whereas the NR2B-containing antagonist Ro25-6981 did not.

**Conclusions:**

PCP dysregulates translation, acting through NR2A-containing NMDAR subtypes, recruiting mGluR1/5 signaling pathways, and leading to neural discoordination that is central to the cognitive and sensorimotor impairments.

Phencyclidine (PCP) is a potent uncompetitive antagonist of glutamatergic NMDA receptors (NMDARs) (1,2) with dopamine-mimetic effects at dopamine D_2_ receptors (3, 4, 5). PCP distorts cognition, causes schizophrenia-like symptoms in healthy people, and exacerbates symptoms in patients with schizophrenia (6, 7, 8). One of the most dangerous hallucinogens, it is increasingly used to lace cannabis and other drugs, and emergency department prevalence of PCP intoxication has been rising (9,10). Using rat spatial cognition, hippocampus, and place cells, we found that PCP impairs cognition and discoordinates neural activity within and between brain networks that are central to cognitive control, the ability to judiciously process information for attaining goals (11).

The consequences of PCP are rapid, suggesting that the cognitive effects may be due to ionotropic actions of blocking NMDARs to reduce excitation. It is unclear, however, whether the cognitive and behavioral effects of PCP arise from hypoglutaminergic or hyperglutaminergic effects (12), NMDAR or secondary non-NMDAR actions. Both PCP and ketamine, another uncompetitive NMDAR antagonist, increase extracellular glutamate in the nucleus accumbens and prefrontal cortex (3,13,14). PCP alters inhibitory interneuron function (15, 16, 17), suggesting that it can be disinhibiting. Indeed, uncompetitive NMDAR antagonists including PCP, ketamine, and dizocilpine (MK-801) all produce network discoordination and psychotomimetic effects (13,18).

PCP and ketamine each transiently increase BDNF (brain-derived neurotrophic factor) (19,20), which rapidly stimulates protein synthesis (21), and ketamine increases protein synthesis (22), suggesting that dysregulated translation could contribute to the effects of PCP. We noticed similarities between PCP-induced enhancement of gamma oscillations in the hippocampus local field potential (LFP) (11,23) and enhancement of gamma in mice that lack the translation repressor BC1 RNA when metabotropic glutamate receptor (mGluR)–stimulated protein synthesis was increased (24). We therefore formulated the working metabolic hypothesis that the neural and cognitive discoordination effects caused by PCP arise from excessive protein synthesis.

## Methods and Materials

**Figure 1:**
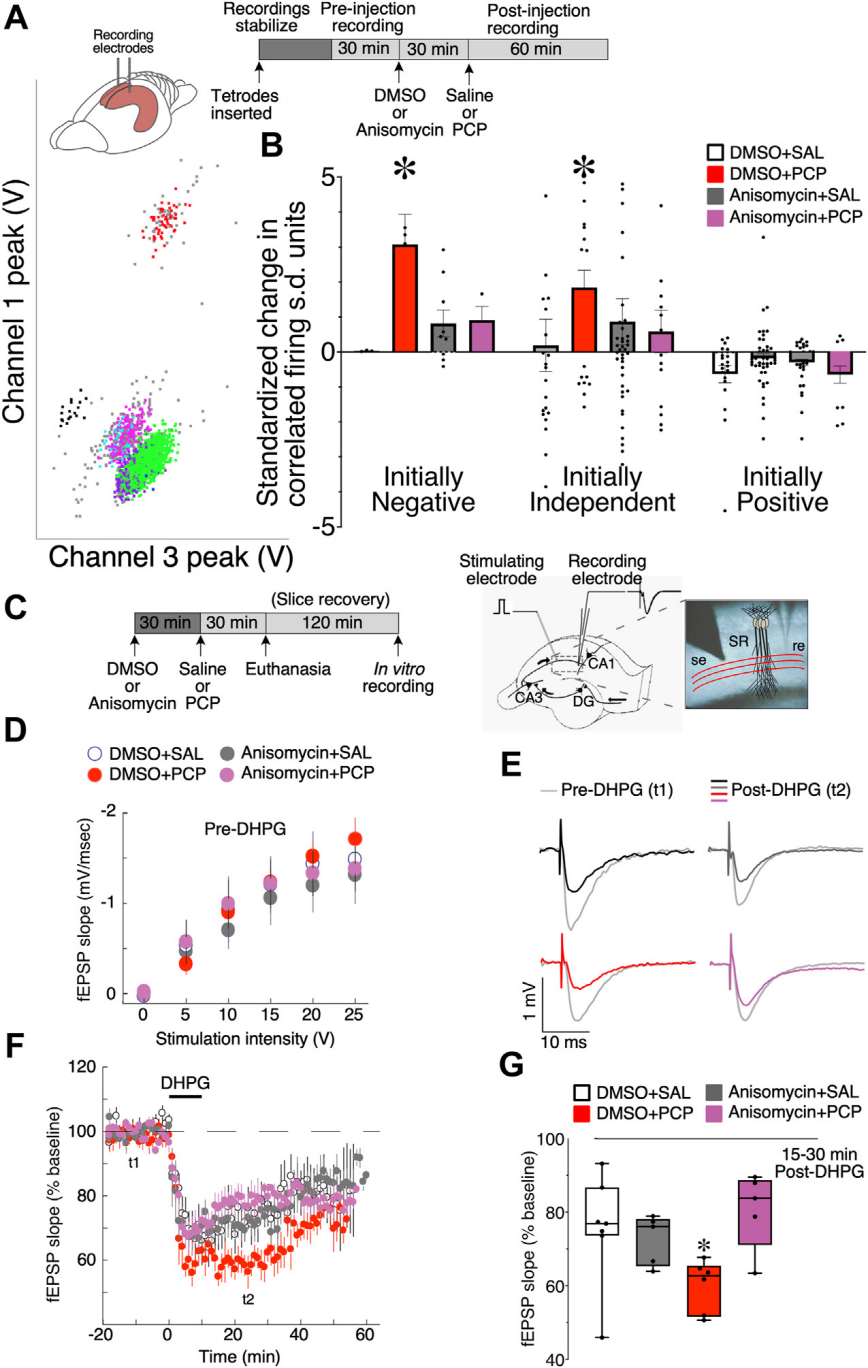
Neural discoordination caused by PCP involves dysregulation of translation. **(A)** Schematic (top right) illustrating the experimental design. CA1 action potentials were recorded from urethane-anesthetized rats using tetrodes and, as shown here, isolated into color-coded single-unit event clusters within the waveform parameter space. **(B)** The change in Kendall’s correlation (τ) from before to after PCP treatment was computed for all pairs of simultaneously recorded pyramidal cells. Cofiring is computed during 30 minutes before and after injection at 250-ms resolution. The cofiring changes are grouped by whether cofiring was significantly negative or positive or independent during preinjection. PCP (8 mg/kg) selectively increases cofiring of initially negatively or independently cofiring cell pairs. This PCP-induced discoordination is blocked by prior administration of anisomysin (60 mg/kg) (cell numbers: DMSO+SAL *n* = 57; DMSO+PCP, *n* = 84; anisomysin+SAL, *n* = 91; anisomysin+PCP, *n* = 31). **(C)** Schematic illustrating the experimental design for fEPSP recordings from CA1 stratum radiatum. Mice received systemic injections before euthanasia and preparation of ex vivo hippocampal slices. The drawing and microphotograph illustrate stimulating and recording electrode locations. **(D)** Before DHPG, the drugs did not affect evoked fEPSP amplitudes (DMSO+SAL, *n* = 7; DMSO+PCP, *n* = 6; anisomysin+SAL, *n* = 5; anisomysin+PCP, *n* = 6). **(E–G)** PCP-enhanced group I mGluR–stimulated, protein synthesis–dependent synaptic plasticity, measured by 50 μM DHPG-stimulated long-term depression. Anisomycin pretreatment prevented mGluR–long-term synaptic depression enhancement by PCP. **(E)** fEPSP examples before (t1 = 10 minutes) and after (t2 = 25 minutes) DHPG. **(F)** Time course of the average change in fEPSP slope. **(G)** Average change between 15 and 30 minutes after DHPG. ∗*p* < .03 relative to DMSO+SAL. DG, dentate gyrus; DMSO, dimethyl sulfoxide; fEPSP, field excitatory postsynaptic potential; PCP, phencyclidine; re, recording electrode; SAL, saline; s.d., standard deviation; se, stimulating electrode; SR, stratum radiatum.

**Figure 2:**
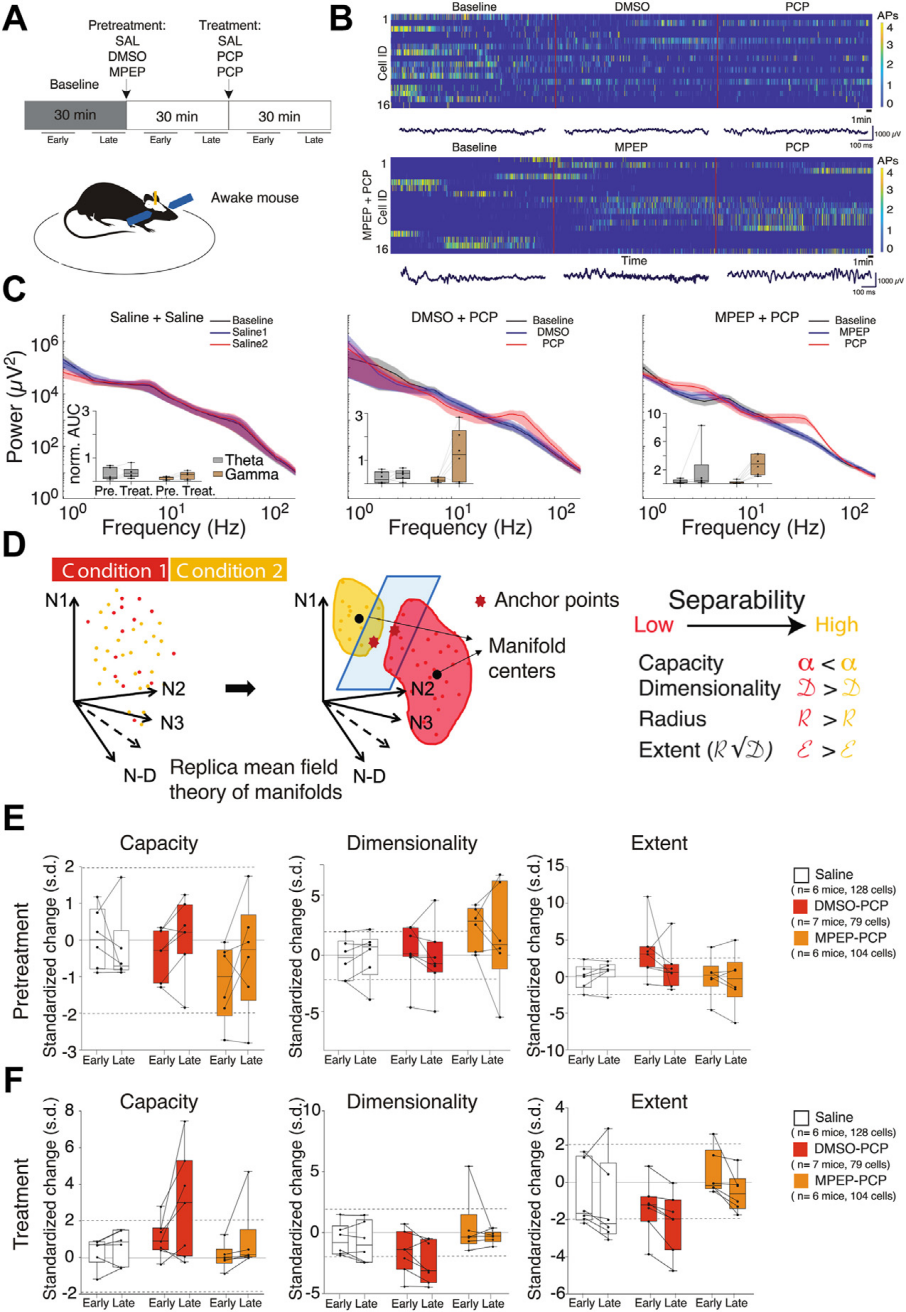
PCP changes the representational geometry of neural population discharge. **(A)** Experimental design. Early: 5–15 minutes; Late: 20–30 minutes after SAL/PCP injection. **(B)** Example recordings of CA1 single unit ensembles and local field potential across the baseline, pretreatment, and treatment 30-minute epochs. **(C)** Average power spectra during baseline, pretreatment, and treatment, with insets that compare the spectral power changes due to pretreatment and treatment on theta and gamma power relative to baseline. **(D)** Drawing depicting the replica mean field theory used to estimate measures of representational geometry in population dynamics. **(E, F)** Measures of 3 geometric properties after pretreatment with SAL, DMSO, or MPEP **(E)** and after subsequent treatment with SAL or PCP **(F)** indicate that ensemble discharge is unchanged by pretreatment, becomes more compact after PCP, but the change is blocked by MPEP pretreatment. Standardized changes are measured in s.d. units computed from the 15 2-minute samples before the pretreatment or treatment manipulations. Dashed lines indicate 2 s.d. estimates of significant differences caused by pretreatment or treatment. AUC, area under the curve; DMSO, dimethyl sulfoxide; N-D, neural dimensions; norm., normalization; PCP, phencyclidine; Pre., pretreatment; SAL, saline; s.d., standard deviation; Treat., treatment.

**Figure 3:**
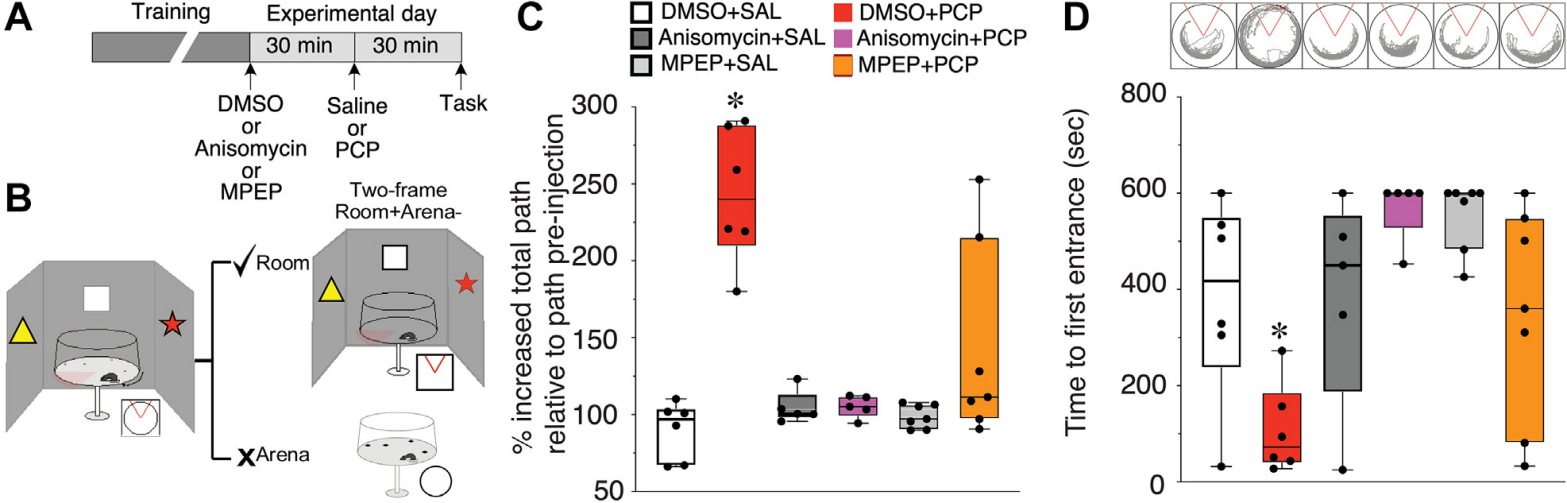
PCP impairs familiar active place avoidance cognitive behavior by dysregulating translation. **(A)** Schematic illustrating the experimental protocol to test whether pretreatment with anisomycin or MPEP blocks PCP impairment of familiar active place avoidance (DMSO+SAL, *n* = 6; DMSO+PCP, *n* = 6; MPEP+SAL, *n* = 7; MPEP+PCP, *n* = 7; anisomysin+SAL, *n* = 5; anisomysin+PCP, *n* = 6). **(B)** Schematic illustrating the task concept and environment. **(C)** Both anisomycin and MPEP pretreatments reduce PCP-induced hyperactivity. **(D)** PCP impairs well-learned active place avoidance in mice. The impairment is prevented by anisomycin or MPEP pretreatment. Inset shows path during test trial for representative mice. ∗*p* < .05 relative to all groups. DMSO, dimethyl sulfoxide; PCP, phencyclidine; SAL, saline.

**Figure 4:**
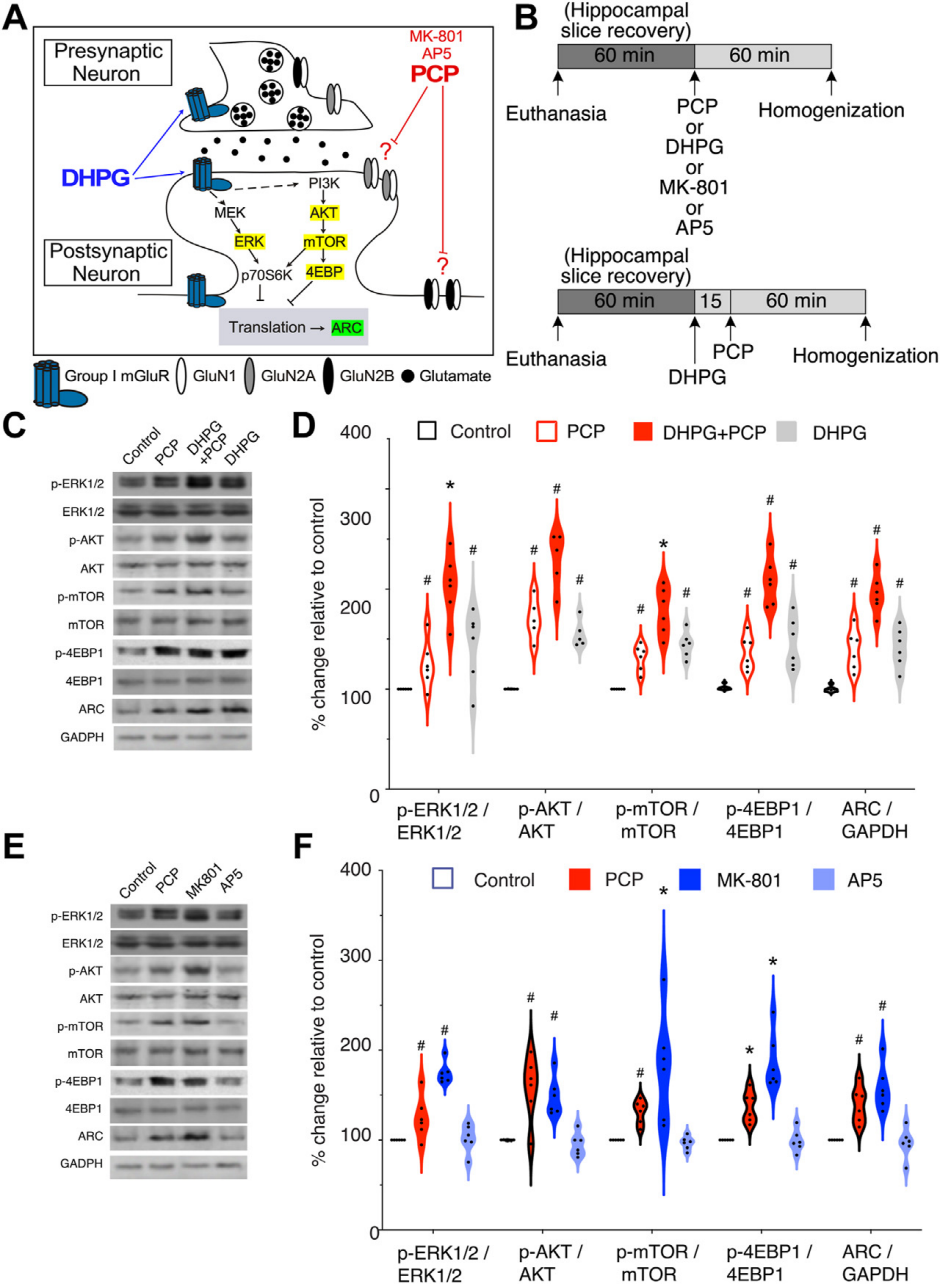
PCP and MK-801 as well as group I mGluR stimulation activates translation, whereas AP5 does not, as measured by expression of the translation machinery components ERK1/2, Akt, mTOR, 4EBP1, and ARC protein. **(A)** Schematic of known and unknown interactions among translation molecular machinery at a CA1 glutaminergic synapse. **(B)** Schematic experimental design. **(C)** In vitro effects of PCP on translation machinery in hippocampus were measured by Western blot. *n* = 6 per group. **(D)** Violin plots show that PCP increased activation of translation machinery proteins. **(E)** Western blot analysis comparing treatment by PCP, MK-801, and AP5. The same control and PCP blots are in panels **(C)** and **(E)**. **(F)** Translation was stimulated by PCP and MK-801, but not AP5. Error bars represent ± SEM. Significant effects were confirmed by 1-way analysis of variance. ^#^*p* < .01 relative to control group; ∗*p* < .01 relative to all groups. ERK1/2, extracellular signal-regulated kinase 1/2; mTOR, mechanistic target of rapamycin; p, phosphorylated; PCP, phencyclidine.

**Figure 5:**
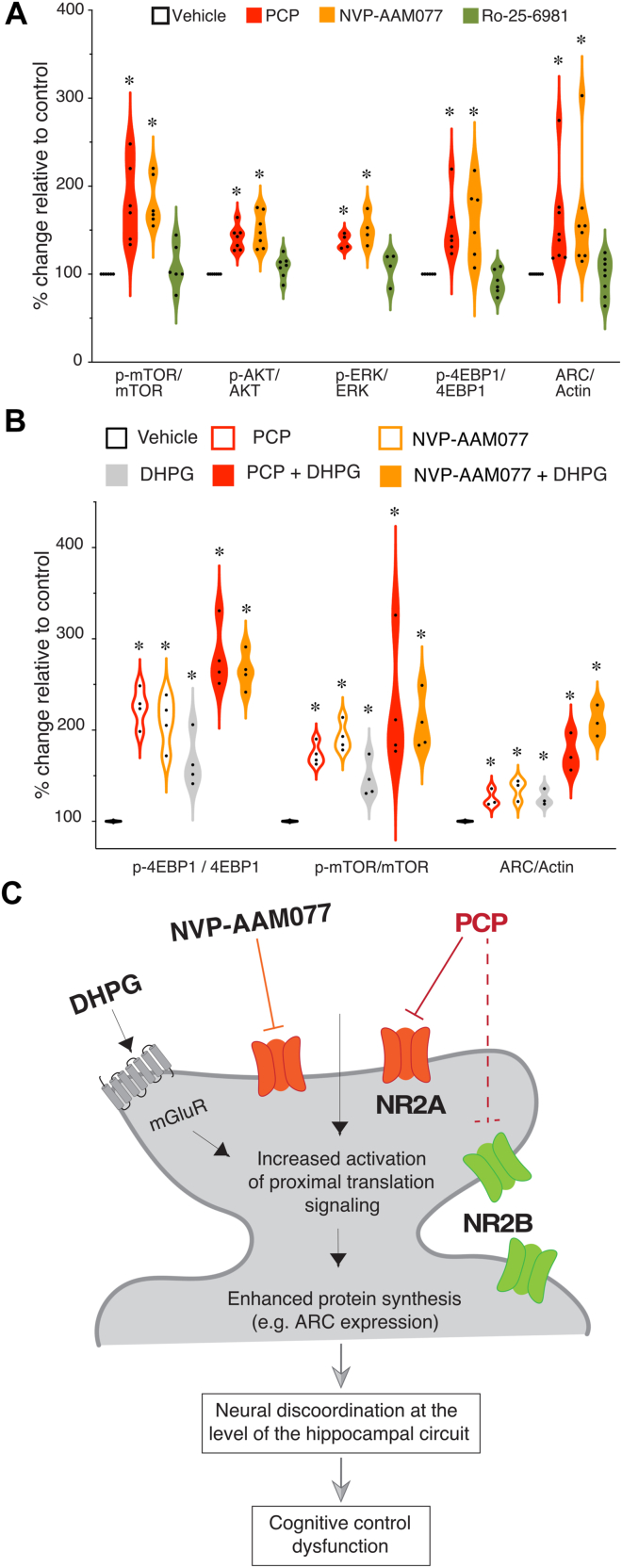
DHPG exacerbates effects of both PCP and the NR2A-containing NMDA receptor antagonist NVP-AAM077. **(A)** PCP and NVP-AAM077 but not Ro-25-6981 increase phosphorylation of the translation-relevant proteins relative to their total expression. **(B)** Similar to PCP+DHPG, cotreatment with NVP-AAM077 and DHPG activates the translation machinery more than single treatments. Each experimental sample was tested twice. **(C)** Scheme summarizing the findings and proposed mechanism of acute PCP intoxication. Similar to NVP-AAM077, PCP predominantly blocks NR2A containing receptors in hippocampal spines, causing increased activation of translation signaling and ARC expression, which is exacerbated by mGluR activity. We suggest that the PCP-induced increase in mGluR-dependent protein synthesis contributes to the neural discoordination observed at the level of the hippocampus circuit and the cognitive control dysfunction, measured using the active place avoidance paradigm. ERK1/2, extracellular signal-regulated kinase 1/2; mTOR, mechanistic target of rapamycin; p, phosphorylated; PCP, phencyclidine.

All methods have been published (11,21,25, 26, 27, 28) and are provided in Supplemental Methods and Materials. Dorsal hippocampus in vivo LFP and pyramidal cell discharge and evoked-potential population responses in ex vivo hippocampal slices were analyzed for Figures 1 and 2, active place avoidance open field behavior was analyzed for Figure 3, and protein synthesis was analyzed for Figures 4 and 5. Comparisons were considered statistically significant when *p* values were < .05; test statistics and degrees of freedom are provided in addition to *p* values.

## Results

### PCP-Induced Neural Discoordination Is Blocked by the Protein Synthesis Inhibitor Anisomycin

The discharge coordination within an ensemble of neurons is estimated by the distribution of correlations between the spike trains of pairs of cells, which in hippocampus CA1 are cell pair specific and stable (11,26). We previously reported that in freely behaving rats, PCP discoordinates CA1 network discharge, selectively increasing discharge correlations among weakly correlated cell pairs (11). The metabolic hypothesis that the neural and behavioral discoordination effects of PCP are caused by dysregulated translation predicts that the discoordinating effects of PCP will be blocked by pretreatment with the protein synthesis inhibitor anisomycin (Figure 1A).

Anisomycin (60 mg/kg intraperitoneal [i.p.] injection) pretreatment did not change single unit discharge or neural coordination, but it blocked the neural discoordination induced by 5 mg/kg of PCP that was selective to initially negatively or independently cofiring cell pairs, but not positively correlated cell pairs (Figure 1B). The effects of anisomycin pretreatment, PCP treatment, and baseline cell pair category were evaluated by 3-way analysis of variance (ANOVA). The effects of pretreatment cell pair category (*F*_2,241_ = 7.60, *p* = 6.3 × 10^−4^) and the interaction between anisomycin and PCP treatment (*F*_1,241_ = 4.11, *p* = .04) were significant. Post hoc tests indicated that only PCP alone increased cofiring. These data replicate prior observations in freely behaving rats and extend them to an anesthetized condition where PCP-induced hyperactivity cannot contribute to the PCP-induced changes in cofiring. These data are the first indication that blocking protein synthesis might be sufficient to prevent the neural discoordination caused by PCP and therefore that dysregulated protein synthesis could be a mechanism by which PCP causes neural discoordination. We note that anisomycin may cause effects other than inhibition of protein synthesis [reviewed by Gold (29)], which include activation of the MAPK (mitogen-activated protein kinase) pathway (30), increased release of a variety of neurotransmitters (31), and even silencing of neurons (32). Although control experiments demonstrate that our administration of anisomycin does not silence neurons, the effects of anisomycin cannot be attributed only to protein synthesis inhibition.

### PCP Potentiates Protein Synthesis–Dependent Group I mGluR-Stimulated Long-term Depression

We sought additional evidence of whether PCP-dependent changes of neurophysiology are mediated by dysregulation of translation. The group I mGluR agonist DHPG produces robust, protein synthesis–dependent long-term depression (LTD) at Schaffer collateral CA1 synapses in hippocampal slices (33,34), which is exaggerated by loss of the negative regulators of translation FMRP (fragile X messenger ribonucleoprotein) (35) or BC1 RNA (25). If PCP dysregulates synaptic plasticity in a similar way, the in vivo PCP treatment should also enhance mGluR-stimulated LTD. Mice were pretreated with anisomycin (60 mg/kg i.p.) or vehicle (dimethyl sulfoxide [DMSO]) 30 minutes before PCP (8 mg/kg i.p.) or saline injections and sacrificed 30 minutes later (Figure 1C). Hippocampus ex vivo slices were treated with 50 μM DHPG to elicit mGluR-stimulated LTD at Schaffer collateral synapses. Before DHPG, neither anisomycin nor DMSO affected field excitatory postsynaptic potential responses (Figure 1D), but PCP enhanced mGluR-stimulated LTD (Figure 1E–G). The PCP enhancement measured ex vivo was blocked by anisomycin pretreatment in vivo. The interaction between anisomycin and PCP was significant (*F*_1,19_ = 6.75, *p* = .02). Dunnett’s test showed that only the group treated with the combination of DMSO and PCP was significantly different from the DMSO+saline control group (*p* = .03) (Figure 1E–G). The anisomycin+saline group showed strong LTD, indicating that anisomycin effects are complex, certainly when assayed in hippocampal slices more than 3 hours after administration to the mouse. There is also evidence of different early and late effects of mGluR1-mediated signaling, which loses its ability to stimulate the neuroprotective, LTD-like mechanisms that require activation of the PI3K-Akt-mTOR signaling pathway (36). Nonetheless, these findings provide additional evidence that motivates investigating whether PCP may stimulate translation and reveal an interaction with group I mGluRs.

### PCP Changes Gamma Power and Representational Geometry of CA1 Population Discharge

We examined the effect of PCP on LFPs and the representational geometry of concurrent CA1 discharge and whether the change is blocked by the group I mGluR5 antagonist MPEP. CA1 LFPs and ensemble discharge were recorded from head-fixed mice running on a wheel (Figure 2A, B). Neither 4- to 8-Hz theta (*F*_2,15_ = 0.13, *p* = .9) nor 30- to 100-Hz gamma (*F*_2,15_ = 0.43, *p* = .7) spectral power was altered by pretreatment with saline, DMSO, or MPEP. Subsequent treatment with PCP did not alter theta compared with saline (*F*_2,15_ = 1.02, *p* = .4), but PCP increased gamma power, and MPEP pretreatment exaggerated the effect (*F*_2,15_ = 9.01, *p* = .003).

CA1 ensemble discharge was investigated using replica mean field theory (37), within which we computed measures of representational geometry that characterize the linear separability of manifold organization of the population activity temporal dynamics (Figure 2D). Pretreatment with saline, DMSO, or MPEP could not be distinguished. The 2-way treatment × time ANOVA confirmed no significant effects of treatment (*F*_2,32_s ≤ 2.53, *p*s > .1) on the replica manifold measures (capacity, dimensionality, and extent). The effects of time (*F*_1,32_s ≤ 0.71, *p*s > .4) and the treatment × time interactions (*F*_2,32_s ≤ 0.82, *p*s > .5) also were not significant. Consistent with PCP increasing correlated activity (Figure 1B), PCP increased the compactness of the population discharge (Figure 2F); representational capacity increased >2 standard deviations (*p* < .05) 20 to 30 minutes, but not 5 to 15 minutes, after PCP injection, whereas MPEP pretreatment (70 mg/kg i.p.) prevented the PCP-induced increase. Two-way treatment × time ANOVA confirmed significant effects of the treatment on capacity (*F*_2,32_ = 3.73, *p* = .03; PCP > saline = MPEP+PCP), dimensionality (*F*_2,32_ = 5.10, *p* = .01; PCP < saline = MPEP+PCP), and extent (*F*_2,32_ = 3.70, *p* = .04; PCP < MPEP+PCP). However, effects of time (*F*_1,32_s < 3.5, *p*s > .07) and the treatment × time interactions (*F*_2,32_s < 0.79, *p*s > .5) were not significant. These findings in behaving mice confirm that group I mGluR antagonism can prevent PCP-induced discoordination.

### Posttraining Impairment of Active Place Avoidance by PCP Depends on Group I mGluR–Stimulated Protein Synthesis

We then investigated whether PCP impairs active place avoidance that requires cognitive control (28,38,39) and whether the impairment requires protein synthesis as predicted by the metabolic hypothesis (Figure 3A). Retention of well-learned room+arena- active place avoidance was tested 30 minutes after systemic administration of PCP (8 mg/kg) or vehicle (Figure 3B). PCP induced hyperactivity (Figure 3C), which was prevented by anisomycin as well as MPEP pretreatment. The main effects were significant as well as the interaction (*F*_2,30_ = 6.24, *p* = .005), and post hoc tests confirmed that the group that received only PCP moved more than the others. PCP did not abolish place avoidance; mice that received DMSO followed by PCP spent 30.1 ± 10.0 seconds in the shock zone, less than chance (100 seconds), but more than the other groups (averages <8 seconds). This difference could be due to multiple factors, including greater extinction learning under PCP. Nonetheless, the PCP-induced differences were also due to impaired memory as well as perhaps impaired sensorimotor integration and cognitive control. The PCP-treated mice walked shorter distances before first entering the shock zone, before they could learn whether the shock was still active (Figure 3D). The impairment was attenuated by pretreatment with anisomycin (60 mg/kg) as well as MPEP (70 mg/kg) (Figure 3D). The 2-way ANOVA on the factors of pretreatment (vehicle, anisomycin, or MPEP) and treatment (PCP or vehicle) identified significant main effects as well as the interaction (*F*_2,30_ = 5.95, *p* = .007). Post hoc tests confirmed that the group that received PCP without either anisomycin or MPEP differed from all the others. The same conclusions are supported by comparing the times to first enter the shock zone, which may be more influenced by hyperactivity (data not shown; 2-way ANOVA interaction: *F*_2,30_ = 7.23, *p* = .002). These findings demonstrate that in addition to neural discoordination, the PCP-induced cognitive and sensorimotor impairments depend on an anisomycin- and MPEP-sensitive process, which is most likely protein synthesis.

### PCP Stimulates Group I mGluR–Dependent Translation

We tested whether PCP activates protein synthesis using Western blot analysis to evaluate the effects of PCP (10 μM), DHPG (100 μM), and the PCP and DHPG combination on activation of the molecular machinery of translation in acute hippocampal slices compared with vehicle control treatment (Figure 4A, B). Because the translation machinery represented by ERK1/2 (extracellular signal-regulated kinase 1/2), mTOR, Akt, and 4EBP1 is active when these proteins are phosphorylated, we measured the ratio of phosphorylated to total protein as an indicator of increased activity of these regulatory proteins (40). ARC, the immediate early gene product, was measured as a downstream indicator of increased translation (41,42). One-way ANOVAs, followed by Tukey post hoc tests when appropriate, were performed to compare the drug effects on each molecule. The drugs increased phosphorylation of ERK1/2, Akt, mTOR, and 4EBP1 and increased ARC (*F*_3,20_s ≥ 15.3, *p*s < 10^−5^) Figure 4C, D). Post hoc tests showed that compared with vehicle control, DHPG treatment significantly increased phosphorylation of the translation machinery proteins, as expected. Similarly, PCP enhanced the phosphorylation of translation machinery proteins that reached significance for Akt, mTOR, and 4EBP1 and was marginal for ERK1/2 (one-tailed *p* = .05). Combined PCP and DHPG treatment further upregulated the phosphorylation of the same molecules beyond the phosphorylation levels observed with single PCP or DHPG treatments (*p*s < .01). Complementing activation of the translation machinery, ARC protein levels were significantly upregulated by individual PCP (*p* < 10^−3^) and DHPG (*p* < 10^−3^) treatments compared with the vehicle control, and the combined treatment of PCP and DHPG resulted in further increased ARC beyond the levels observed with single treatments (*p*s < 10^−3^). These data directly confirm that PCP interacts with group I mGluRs to promote translation.

We compared the PCP findings with related drugs by also testing whether MK-801, another uncompetitive NMDAR antagonist, and AP5, a competitive NMDAR antagonist, also activate the translation machinery and upregulate ARC. The drug effects were significant in all the assays (*F*_4,20_s ≥ 9.85, *p*s ≤ 10^−4^). Similar to PCP, relative to vehicle, MK-801 (25 μM) upregulated the phosphorylation of mTOR, ERK1/2, Akt, and 4EBP1 as well as increased ARC (*p*s ≤ .001) (Figure 4E, F). However, AP5 (50 μM) did not significantly alter the phosphorylation of the analyzed molecules or ARC relative to vehicle (*p*s ≥ 0.7) (Figure 4E, F). We conclude that PCP deregulates translation, similar to another uncompetitive NMDAR antagonist, MK-801, but in contrast to the competitive NMDAR antagonist AP5.

### NR2A-Selective Antagonist NVP-AAM077 Mimics PCP-Induced Activation of Translation

We next examined if PCP might predominantly act through NR2A- or NR2B-containing NMDA receptors by testing whether antagonists of NR2A- or NR2B-containing receptors mimic the effects of PCP on translation. Acute hippocampus slices were treated with PCP (10 μM), the NR2A-selective blocker NVP-AAM077 (43), or the NR2B-selective blocker Ro25-6981 (44) (each of the blockers at 0.5 μM concentrations).

Compared with vehicle controls, PCP and NVP-AAM077 caused significant increases in the ratio of phosphorylated to total protein for ERK1/2, mTOR, Akt, and 4EBP1 (all *n* = 6–8, *F*_3,28_s > 5.76, *p*s ≤ .003) (Figure 5A). The increased ratios did not differ between PCP and NVP-AAM077 (*p*s ≥ .3). In contrast, compared with vehicle, Ro25-6981 did not have any significant effects on the above-mentioned proteins (*p*s ≥ .4). The total protein levels of ERK1/2, mTOR, Akt, and 4EBP1 were not altered by any of the in vitro treatments (data not shown). PCP and NVP-AAM077 each significantly increased ARC compared with vehicle (*p*s ≤ .003), whereas Ro25-6981 did not (*p* = .4) (Figure 5A).

### Molecular Effects of PCP and NVP-AAM077 Are Exacerbated by DHPG

Because NMDARs and group I mGluRs colocalize (45,46), we tested whether both PCP and NVP-AAM077 act through the group I mGluR signaling pathway to stimulate translation. We compared the effect of the two NMDAR antagonists on translation in the presence of the group I mGluR agonist DHPG (100 μM), which we had confirmed combines with PCP to enhance mGluR-stimulated synaptic plasticity that requires translation (Figure 1D–G). The effects of the antagonists all were significant (*F*_2,12_s > 14.3, *p*s ≤ .0002; PCP = NVP-AAM077 > vehicle) as was the effect of DHPG (*F*_2,12_s > 7.6, *p*s ≤ .01). The antagonist × DHPG interaction was significant only for the effect on ARC (*F*_2,12_s > 5.2, *p* = .02; PCP+DHPG = NVP-AAM077+DHPG > PCP = NVP-AAM077 > vehicle). In summary, DHPG treatment alone increases the phosphorylation of 4EBP1 and mTOR and enhances ARC expression, as expected (Figure 5B), whereas cotreatment of PCP or NVP-AAM077 with DHPG further enhances translation signaling.

## Discussion

### Main Findings

We used multiple assays at distinct levels of biological organization and pharmacological manipulations to discover that PCP disrupts neural coordination and cognitive control by dysregulating translation. First, PCP discoordinated the temporal organization of spiking in urethane-anesthetized rats by coactivating anti-cofiring cell pairs that were unlikely to discharge together before PCP (Figure 1). PCP also increases CA1 gamma oscillations (Figure 2C) (11) and discoordinates CA1 neural population discharge from the awake mouse CA1, resulting in more stereotyped and distinctive neural population dynamics (Figure 2). These findings replicate and extend previous findings in freely behaving rats, as PCP-induced hyperlocomotion and other behavioral confounds cannot account for the present electrophysiological findings (11). The PCP-induced neural discoordination is similar to the discoordination effects of MK-801 (47) as well as the effects of hippocampal disinhibition by tetrodotoxin inactivation of the contralateral hippocampus (26). Second, PCP also impaired a familiar active place avoidance task that requires cognitive control demonstrated behaviorally (Figure 3) as well as with electrophysiology and calcium imaging concurrent with behavior (28,38,39). This extends to mice previous observations in rats (11). Third, anisomycin and MPEP pretreatments prevented the electrophysiological, cognitive behavioral, and sensorimotor effects of PCP. We also demonstrate that PCP enhances group I mGluR–stimulated LTD, a protein synthesis–dependent type of synaptic plasticity, whereas PCP itself does not (Figure 1). These findings argue that the cognition-impairing effects of PCP arise from dysregulated group I mGluR–stimulated protein synthesis. Fourth, PCP increased proximal, translation signaling in both rat (Figure 4) and mouse (Figure 5) hippocampus as indicated by enhanced ARC expression and increased phosphorylation of ERK1/2, Akt, mTOR, and 4EBP1, consistent with either anisomycin or MPEP preventing PCP-induced neural discoordination (Figures 1 and 2). Finally, mimicry experiments indicate that PCP-induced activation of translation can be the result of the antagonist action of PCP on NR2A-containing NMDARs leading to group I mGluR stimulation (Figures 4 and 5).

### Comparison With Neocortical Effects of NMDAR Antagonists: The Discoordination Hypothesis

PCP and MK-801 as well as contralateral hippocampal inactivation with tetrodotoxin all cause the same specific pattern of neural discoordination as well as a specific cognitive behavioral deficit in the use of established spatial memories, rather than in place learning or information storage per se (11,26,48, 49, 50). These observations are predicted by the discoordination hypothesis (51,52), which asserts that psychosis-related cognitive deficits, such as impaired cognitive control, arise because processing multiple streams of information is corrupted by aberrant coordination of neural activity, in which cells that normally discharge together and cells that normally do not discharge together fail to maintain their appropriate timing relationships, despite maintaining their individual response properties (26,53, 54, 55, 56, 57, 58). The idea derives directly from cell assembly (59) and other population dynamics hypotheses for how information is represented in the brain, which seem valid in hippocampus (38,60, 61, 62, 63, 64, 65, 66). These hypotheses assert that streams of information are represented by the coordinated temporal spiking relationships among cells in distributed representations (67). It follows that temporal discoordination, particularly loss of anti-cofiring (Figures 1B and2), will derange representations and the judicious use of information, predictions that are confirmed by the present findings.

Prior work demonstrated electrophysiological discoordination after PCP and other NMDAR antagonists. Similar to the present work in hippocampus, ketamine and MK-801 each increased gamma band power (68) as well as increased correlated discharge in neocortex (18), which in the case of ketamine is accompanied by diverse region-specific increases and decreases in metabolism assessed by 2-deoxyglucose imaging (69) and increased glutamate and dopamine release (13). The discoordinating effects of MK-801 on prefrontal cortical neurons is likely due to drug-dependent decreases of inhibitory control and consequent increases of excitatory firing (70,71), whereas we did not see increases in discharge rates in our studies of hippocampus, likely due to especially strong firing rate homeostatic mechanisms in hippocampus (11,72). We did not assess whether the effects of PCP were reversible, but the neuronal discoordination lasts < 1 hour (11); the metabolic effects are nonetheless likely to have produced enduring effects, which in the case of attenuated LTP have been described to persist for weeks following a single dose of MK-801 (73). The metabolic effects of PCP are likely due to antagonism of NR2A-containing NMDA receptors. This does not imply that the electrophysiological effects are also due to NR2A antagonism, although NMDAR deletion in parvalbumin-expressing neurons is sufficient to express enhanced gamma (74). Low doses (≤ 10 mg/kg) of MPEP are reported to decrease prefrontal cortical neuronal discharge (75), but we did not observe such effects of high-dose MPEP in hippocampus CA1; the electrophysiological drug effects are likely to be subregion specific and dependent on the region’s particular network properties.

### Mechanism of PCP-Induced Cognitive Impairments

PCP-induced discoordination is rapid, within a minute in the awake rat (11), suggesting ionotropic effects consistent with alteration of interneuron function (15,16,70), but as the present findings demonstrate, PCP effects are also metabolic, which can also be rapid, within 5 minutes (76,77). PCP transiently increases BDNF (19), which rapidly stimulates protein synthesis via the mTOR pathway (Figures 4 and 5) (21). Impairment of cognition due to PCP also appears to be related to dysregulation of mGluR-stimulated translation because the PCP effects were blocked by either anisomycin or MPEP and are exacerbated by group I mGluR stimulation (Figures 1, 3, and 4). The PCP effects can be through both the mGluR-MEK/ERK and the mGluR-mTOR translation pathways (Figures 4 and 5), although this requires further investigation; ketamine and MK-801 affect both pathways (22,78, 79, 80). While controversy persists over which translation pathways are more relevant for ketamine’s effects (20,22,81,82), the present findings with AP5, MK-801, Ro25-6981, and NVP-AAM077 demonstrate that different NMDAR antagonists have different effects on translation and so different potential for intoxication and therapy.

PCP enhanced mGluR-dependent translation to produce cognitive impairment. Figure 5C summarizes the findings, but there are multiple possibilities for how PCP interacts with mGluR-stimulated translation, and we note that MPEP itself is a weak NMDAR antagonist (83). The most parsimonious possibility is that by blocking NR2A-containing NMDARs, PCP makes more glutamate available to stimulate colocalized mGluRs (45,46), but it is not obvious why uncompetitive open channel blockade by PCP should reduce glutamate binding to NMDARs (84,85). Alternatively, PCP can increase glutamate signaling onto principal cells by preferentially blocking NR2A-containing NMDARs on interneurons (15,16,70) or possibly by interacting with astrocytic gliotransmission that is sensitive to group I mGluR stimulation (86,87). Aberrantly increased glutamate signaling would increase mGluR stimulation, initiating a molecular cascade that increases translation at synapses causing a dysregulation of excitation and inhibition (88). A weak prediction of this model is increased principal cell firing, which is not observed (Figure 2B) (11). However, the observation may be the result of tight coupling between excitation and inhibition and mechanisms of firing rate homeostasis in hippocampus (26,72).

NMDAR and mGluR interactions are likely to contribute to the interplay between PCP and mGluR signaling, which is complex and all the more so because mGluR3 activation potentiates mGluR5 activity (89). Chemical stimulation of NMDARs enhances repression of translation by microRNAs (90). By blocking NR2A-containing NMDARs, PCP would derepress translation by this mechanism. It is also likely that mGluR stimulation will itself derepress translation because DHPG treatment of primary neuronal cultures stimulates presynaptic mGluRs, resulting in AMPA receptor endocytosis, which has been shown to result in enhanced translation (90, 91, 92, 93). These NMDAR and mGluR mechanisms of stimulated translation may account for the present findings that PCP blockade of NMDARs promotes translation and that the effect of mGluR stimulation by DHPG is additive, whereas mGluR5 antagonism attenuated the electrophysiological (Figure 2) and behavioral (Figure 3) effects of PCP. PCP also enhanced ARC synthesis, which is implicated in downregulation of surface-bound AMPA receptor and LTD (76,94). However, because the effects of PCP intoxication on cognitive and neural representation are transient, lasting approximately 1 hour (11), persistent changes in synaptic plasticity are unlikely to explain our observations, but may account for the derangements that follow repeated exposure (95, 96, 97).

The prevention of PCP-induced hyperlocomotion and active place avoidance impairments by high dose of MPEP (Figure 3) contradicts reports that group I mGluR antagonists exacerbate the effects of PCP and related NMDAR antagonists (98, 99, 100, 101, 102, 103) and reports that positive allosteric modulators have either beneficial or no effects (100,104). Most, but not all, of these studies assayed effects on sensorimotor gating, whereas we focused on cognitive effects. Nonetheless, assessment differences are insufficient to explain the outcome differences because we also observed that MPEP prevents PCP-induced hyperlocomotion (Figure 3C). Prior studies administered mGluR5 antagonist doses up to 10 mg/kg, whereas we pretreated the mice with 70 mg/kg of MPEP 30 minutes before PCP to ensure effectiveness during training despite the relatively rapid metabolism of MPEP in mice (105,106). In pilot work, we had observed that lower doses of MPEP did not attenuate PCP-induced hyperactivity and that sometimes PCP could transiently cause hyperactivity immediately after it was administered, even after the pretreatment with 70 mg/kg of MPEP. The present findings are clear and robust at the levels of ex vivo and in vivo electrophysiology and cognitive behavior, demonstrating that mGluR5 antagonists can be procognitive, apparently by dampening translation that PCP can stimulate. These findings may be relevant for evaluating use of PCP in procognitive antipsychotic drug development (see Supplemental Discussion). The PCP metabolic effects suggest a potential strategy for reducing the effects of PCP intoxication, which appears distinct from the group II mGluR agonist strategies that were once considered for procognitive antipsychotic development (107, 108, 109).

## Supplementary Material

Supplementary Material

Key Resources Table
