# Supplementary material for: Phencyclidine Disrupts Neural Coordination and Cognitive Control by Dysregulating Translation
Source: Biol Psychiatry Glob Open Sci. 2023 May 31;4(1):252–63. doi: 10.1016/j.bpsgos.2023.04.009 (PMC10829677; doi:10.1016/j.bpsgos.2023.04.009)
Supplement: Supplementary Material [file mmc1.pdf]

SUPPLEMENTARY INFORMATION

Phencyclidine Disrupts Neural Coordination and Cognitive Control by Dysregulating Translation

Park *et al.*

SUPPLEMENTAL MATERIALS AND METHODS

All behavioral and electrophysiological methods have been published elsewhere (1-6). Dorsal hippocampus pyramidal cell discharge and evoked-potential population responses were analyzed for Figures 1 and 2, behavior was analyzed for Figure 3, and protein synthesis was analyzed for Figures 4 and 5.

Drugs

Phencyclidine (PCP, 1-(1-Phenylcyclohexyl)piperidine hydrochloride, pH 7.4; Sigma, St. Louis, MO, Cat. No. P3029) and 2-methyl-6-(phenylethynyl)pyridine (MPEP; Tocris, Ellisville, MO, Cat. No. 1212/10), solutions were made fresh in sterile saline before use. Anisomycin (Sigma, St. Louis, Cat. No. A9789), solution was made fresh in DMSO before use. (S)-3,5-Dihydroxyphenylglycine, (DHPG, Tocris, Ellisville, MO, Cat. No. 0805), D-2-amino-5-phosphonopentanoate (D-AP5, Tocris, Ellisville, MO, Cat. No. 0106), and Dizocilpine ((+)-MK801, Sigma-Aldrich, St. Louis, MO, Cat. No. M107), Ro 25-6981 maleate (R&D Systems, Cat.No. 1594), and NVP-AAM077/PEAQX (Sigma, Cat.No. P1999) were dissolved in sterile water.

Animals

All animals were maintained on a 12:12 light-dark cycle. Adult male Long-Evans rats and C57BL/6 mice were 2-3 months during the experiments. All procedures conformed to institutional and NIH guidelines for the treatment of vertebrate animals and were approved by the SUNY Downstate and NYU Institutional Animal Care and Use Committees.

In vivo electrophysiology – Acute recordings under anesthesia

The *in vivo* methods to record single unit discharge from urethane-anesthetized rats have been described in prior work (2). Briefly, rats were anesthetized with urethane (1.2g/kg i.p.) then

mounted in a stereotaxic frame with precision micromanipulators (TSE Systems GmbH, Bad Homburg, Germany). The scalp was resected and crainotomies were performed at AP -3.8 mm, ML ±2.5 mm relative to bregma to provide electrode access to the dorsal hippocampus. The dura was cut, and tetrode-configured electrodes made from 4 twisted 25-μm nichrome wires (impedance 50-200 kΩ), were lowered to the recording targets using the stereotaxic micromanipulators, guided by electrophysiological landmarks in the local field potentials and single unit activity. Data collection began once stable single unit ensemble activity could be isolated. A baseline recording was made for at least 30 minutes and then the rat received an i.p. injection of the 5 mg/kg PCP or vehicle solution. Recordings continued at least one hour after the injection.

Recording setup

Extracellular, 2-ms tetrode action potential waveforms were buffered by a custom pre-amplifier, band-pass filtered (low-cut frequency 300 or 360 Hz, hi-cut frequency 5-10 kHz), amplified (5,000-10,000 times), and digitized (32 or 48 kHz). The digital signals were stored in real-time using either custom software (AcX, A.A. Fenton) or a commercial system (dacqUSB, Axona Ltd., St. Albans U.K).

The neuronal activity was recorded under anesthesia, for at least 30 min in the pre-injection period. Afterwards the rats received a systemic injection of the control, or behaviorally-effective dose of PCP. Post-injection recordings lasted at least 60 min. All data were analyzed off-line. The data recorded in the 30-min pre-injection and the first 30-min of post-injection interval were analyzed, during which PCP drug status was in an effectively steady state (6).

Single unit identification

The action potentials emitted by different cells were discriminated off-line using custom waveform parameter clustering software (Wclust, A.A. Fenton). Each action potential waveform was characterized by parameters that included the positive and negative peak voltages on each tetrode wire, the voltage at user-selected times relative to the spike onset, the waveform energy, principal components, and others. Single units were classified as those waveforms that formed clusters in the waveform parameter space according to quantitative criteria of isolation from all other spikes ($Iso_{BG} \geq 4$) and isolation from the most similar cluster of spikes ($IsoI_{NN} \geq 4$) (7).

In vivo electrophysiology – Recordings in head-fixed behaving mice

Mice under isoflurane anesthesia were surgically prepared for head-fixed recordings. A titanium head plate was attached to the mouse's skull using dental cement and the exposed skull was covered with KwikSil, a low toxicity adhesive (World Precision Instruments, Sarasota, FL) and protected by attaching a plastic cup. All mice were allowed at least 1 week to recover. A secondary surgery was performed immediately before the experiment. The plastic cup and KwikSil were removed, and a craniotomy was made at 1.85 AP, ± 1.20 ML relative to bregma to enable electrode placement targeting dorsal hippocampus. After recordings the KwikSil protective cup assembly was reattached to prevent infection.

Once the mouse was head-fixed by the titanium plate, A Neuronexus (Ann Arbor, MI) silicon probe was positioned to record from CA1 using a Kopf stereotaxic arm. Signals were filtered between 300 Hz and 10 kHz and sampled at 30 kHz for single unit recording and filtered between 0.1 Hz and 1 kHz and digitized at 2 kHz to record the concurrent LFPs.

LFPs at the CA1 recording sites were averaged for analysis. Powerspectra were computed using the pwelch() function in MATLAB. Theta (4 - 8 Hz) and gamma (30 - 100 Hz) band power were computed, and for comparisons, normalized by the spectra computed from the preceding baseline

recording. The normalized spectra were compared across pretreatment with saline, DMSO and MPEP, and compared separately across the saline-saline, DMSO-PCP, and MPEP-PCP treatment groups.

Single units were sorted using a published open-source algorithm Kilosort2 (8) that takes advantage of GPU processing to improve algorithm performance. After automated clustering of the data, only units with < 20% estimated contamination rate with spikes from other neurons that were computed from the refractory period violations relative to expected. We also excluded units with non-characteristic or noisy waveforms to identify single units.

Classification of cell types

Single units were classified as complex-spike or theta cells according to published criteria (9-11). Complex-spike cells appear to be pyramidal cells, whereas theta cells are likely local interneurons (12). Pyramidal cells were judged to have long-duration waveforms (> 250 μs), low discharge rate (< 2 AP/s) and a tendency to fire in bursts (peak inter-spike interval < 10 ms). Interneurons had short-duration waveforms (< 250 μs), high discharge rate (> 2 AP/s), and did not tend to fire in bursts.

Functional coupling of cell pairs

The functional coupling of spike trains from pairs of cells was estimated using Kendall's correlation (13), which was computed as previously described (2). The correlation was computed between the time series of spike counts from the two cells. The time series was generated by counting the number of spikes the cell fired during each 250-ms interval.

Geometric properties of high-dimensional neural manifolds

The replica mean field theory of manifolds computes the geometric properties of neuronal population activity without assumptions on the topology or dimensionality of the high dimensional neural data (14). The replica-manifold theory assumes that neural manifolds may be defined by D+1 coordinates, such that one coordinate is needed to describe the location of the neural manifold center, and D coordinates to account for the axes that define the manifold variability. Ensemble neural activity organizes on P manifolds sampled M times along N different feature dimensions, i.e., individual cells. In this analysis, there are P manifolds that correspond to P recordings of an animal in different conditions within a recording.

We use three geometric properties to characterize high-dimensional neural data: capacity, dimensionality, and extent. Classification capacity, α_c is defined as the maximum number of manifolds that can be linearly separated given a random assignment of binary labels to manifolds. Capacity can be understood as a high-dimensional measure of overlap between manifolds, a measure of manifold compactness. Similar to the concept of support vectors in linearly separable points, in replica theory, the high dimensional separating hyperplane is defined by a linear combination of what we call manifold anchor points. Anchor points uniquely define the separating plane between manifolds. Anchor points depend on the location and orientation of the manifolds as well as the randomly assigned binary labels. For a given manifold, one can define a statistical distribution of anchor points. From the distribution of anchor points, we can learn about two geometric properties of high dimensional manifolds: the effective radius, R_M, defined as the square root of the total variance of the anchor points, normalized by the average norms of the manifold centroids, and the effective dimension, D_M which captures the spread of the anchor points along the different manifold axes. We further define the manifold total extent, a measure of the space occupied by the high dimensional neural manifold as the mean $R_M\sqrt{(D_M)}$. According to the replica mean field theory of manifolds, larger D_M and $R_M\sqrt{(D_M)}$ of the manifolds are linked to statistically lower linear separability between manifolds, and less distinction among themselves.

Ex vivo hippocampal slice electrophysiology

Mice were systemically pretreated with anisomycin (60 mg/kg) or the DMSO vehicle and 30 min later with PCP or saline. Mice were sacrificed 30 min after the second treatment to obtain hippocampal slices (400 μm). Slices were incubated for 2 hours in oxygenated aCSF (in mM: 125 NaCl, 2.5 KCl, 1 $MgSO_4$, 2 $CaCl_2$, 25 $NaHCO_3$, 1.25 NaH_2PO_4 and 25 Glucose), and then were placed in a submerged chamber subfused with aCSF at 35-36ºC for recording (1, 15). A pair of stimulation (bipolar; FHC & Co, ME, USA) and recording electrodes (borosilicate glass pipette filled with aCSF; 5-10 mΩ) was used to evoke and record field excitatory postsynaptic potentials (fEPSP) at the CA1 *stratum radiatum*. Stimulus-response curves were created by delivering square pulses (50 μsec) at increasing voltages (0-25V). For long-term synaptic depression (LTD) studies, fEPSP responses were set at 40% of the maximum slope amplitude and sampled once per minute. After a stable baseline was established, the group I mGluR agonist DHPG (25 µM) was washed onto the slices for 10 min to induce LTD. LTD slope amplitudes (percentage change from baseline at 15-30 min) between conditions were compared.

Behavior - Active place avoidance task

Setup

An individual mouse was placed on a metal disk-shaped arena (40 cm in diameter). The arena was elevated 76 cm above the floor and centered in 3x4 m^2 room surrounded by opaque curtains and various items (Fig. 3B). The mouse was constrained to remain on the disk by a 40-cm-high transparent wall. The mouse's position was tracked every 33 ms from an overhead camera using digital video spot tracking software (Tracker, Bio-Signal Group Corp., Acton, MA).

The two-frame Room+Arena- task variant was used. The arena rotated at 1 rpm and an unmarked

60° shock zone was fixed at stationary room coordinates. The arena floor was made of parallel metal rods, configured as five electrical poles for delivering a mild electrical shock (0.2 mA, 60 Hz, 500 ms) scrambled across the rods. Under software control, shock was delivered when the mouse was in the shock zone for 500 ms. The shock was repeated every 1.5 seconds until the mouse left the shock zone. To avoid a shock the mouse had to use the relevant stationary distal room cues to localize itself and the positions of the shock zone. Because the arena was rotating the mouse also had to ignore the irrelevant information from the rotating spatial frame. Note that this shock regime is no more stressful than walking freely in the arena (16).

Behavioral Protocol

The goal was to test the effect of PCP on the ability of mice to do active place avoidance after the task had been learned and the memories established, extending the finding that PCP impairs familiar active place avoidance in rat to the mouse (6). The mice were first habituated to the environment during a 10-min trial on the stationary arena with no shock. The shock and arena rotation were then turned on and mice were given three 10-min training trials with 30-min inter-trial intervals for 4 days. On day 5, each mouse received DMSO, MPEP (70 mg/kg), or anisomycin (60 mg/kg) 30 min before either saline or PCP (8 mg/kg). Thirty min after the 2nd treatment, mice were tested in conditions that were identical to the training but without shock.

The time series of the animal’s positions were analyzed offline. The time to first enter the shock zone was measured because it increases as the animals learn to avoid shock and can be interpreted as an index of the ability to retrieve the place avoidance memory. The distance the animals walked was used to estimate locomotion because it can characterize hyperactivity, which is known to be caused by PCP (17).

Protein expression - Western blotting

We used western blot analysis to evaluate the effects of PCP and other drugs on the activation of the protein translation machinery. Specifically, extracellular-signal-regulated kinase (ERK), mammalian target of rapamycin (mTOR), protein kinase B (AKT), and 4E-binding protein (4E-BP1) are involved in proximal translation signaling; ERK is also involved in many other cellular metabolic processes (18). These proteins are active when phosphorylated; therefore, a larger ratio of phosphorylated protein/total protein indicates increased activity of these regulatory proteins and more downstream protein synthesis. Activity-regulated cytoskeleton-associated protein (ARC) expression was measured as a downstream indicator of increased translation. Arc is an immediate early gene; its expression is correlated with neural activity (19).

The effects of the drugs on activating translation were evaluated in both rat and mouse acute hippocampal slices. Treatment chambers for the slices were constructed from 40 mL specimen containers (Simport Scientific), Netwell 24 mm mesh inserts (Electron Microscopy Sciences), and small, sterilizer glass beads (Sigma). Tubing with flow regulators from standard, disposable intravenous sets delivered 95% O_2/ 5% CO_2 to the bottom of each chamber.

Two-month-old rats were decapitated to obtain acute hippocampal slices (400 µm). Brains were extracted and immersed in chilled, oxygenated cutting medium (220 mM sucrose, 20 mM NaCl, 2.5 mM KCl, 1.25 mM NaH_2PO_4, 26 mM $NaHCO_3$, 10 mM glucose, 2 mM ascorbic acid, and 2.5 mM $MgSO_4$). Hippocampi were bilaterally dissected in cold cutting medium. Using a dry McIlwainTM Tissue Chopper (Stoelting Co), 400 µm hippocampal slices were cut and stabilized in chambers containing cutting medium bubbled with 95% O_2, 5% CO_2 for 20 minutes at 35 °C. Slices were transferred and pre-incubated in oxygenated (95% O_2, 5% CO_2) artificial cerebrospinal fluid (aCSF; 125 mM NaCl, 3 mM KCl, 1.25 mM NaH_2PO_4, 26 mM $NaHCO_3$, 2 mM ascorbic acid, 10 mM glucose, 1.5 mM $MgSO_4$, and 2.5 mM $CaCl_2$) at 34°C for 1 h.

Wild-type, male C57/BL6J mice, 12-13 weeks-old, were decapitated to obtain acute 400 μm hippocampal slices prepared similar to those from rats. Slices were transferred to oxygenated aCSF (124 mM NaCl, 2.5 mM KCl, 1.25 mM NaH_2PO_4, 24 mM $NaHCO_3$, 2 mM ascorbic acid, 10 mM glucose, 1.5 mM $MgSO_4$, and 2.5 mM $CaCl_2$) at 35°C before adding pharmacological agents (20).

Drugs were added directly into aCSF for 1 h at the indicated final concentrations: 10 μM PCP, 100 μM (S)-3,5-DHPG, 25 μM MK801 maleate, 50 μM AP5, 0.5 μM Ro25-6981 maleate, and 0.5 μM NVP-AAM077. Slices co-treated with DHPG received pre-treatment for 10 minutes with DHPG followed by addition of the NMDAR antagonist for 1 h.

At the end of the drug and control treatments, slices were collected in 4°C lysis buffer containing protease and phosphatase inhibitors (65 mM Tris-HCl, 10% glycerol, 2% SDS, 5 mM EGTA, 5 mM EDTA, 200 μM Na_3VO_4, 200 μM PMSF, 2 mM NaF, 0.5% Triton, 10 mM Beta-glycerophosphate disodium pentahydrate, 10 mM sodium pyrophosphate, phosphatase inhibitor cocktail (Roche, Cat.No.4906837001) and protease inhibitor cocktail (Roche, Cat.No.4693124001) as described previously (5). Tissue was mechanically homogenized, dry sonicated (Branson Sonifier 250), and then centrifuged at 10,000g x 2 min. The supernatant was denatured for 2 rounds x 5 min in a 95°C heat block. Protein determination was performed with the Micro BCA™ Protein Assay Kit (Pierce, Cat. No.23235). Sample concentrations were adjusted with lysis buffer and then denatured at 95°C for 5 min in 20% loading buffer (10% SDS, 0.3125M Tris HCl pH 6.8, 3.575M beta-mercaptoethanol, 0.05% bromophenol blue in H_2O). Forty μg denatured protein per well was resolved by 8-12% SDS-PAGE homemade gels in parallel with multi-colored molecular weight marker (Bio-Rad Laboratories, Cat.No.1610395). Gels were run at 100V for 1-2 h and transferred overnight at 8-12V, 4°C onto 0.2uM PVDF membranes (GE

Healthcare Biosciences). After transfer, gels were stained for 1 h with Coomassie Blue R-250 0.5% and membranes were stained with MemCode^{TM} Reversible Stain (Pierce, Cat.No.24585) to examine the quality of the protein transfer. Membranes were blocked for 1 h in I-Block^{TM} (Applied Biosystems) protein-based blocking reagent and then incubated with primary antibodies overnight at 4°C. Between primary and secondary antibody incubations, membranes were rinsed three times in 1X Tris buffer solution (TBS) and then washed in I-Block^{TM} 3 x 5 min. Membranes were incubated for 1 h at room temperature in horseradish peroxidase-conjugated secondary antibodies in I-Block^{TM} solution, followed by three rinses and 3 x 5 min washes in 1X TBS. Imaging and detection were performed with ECL Select^{TM} (GE Healthcare, Cat.No.RPN2235) on a C-DiGit^{TM} Blot Scanner (LI-COR, Cat.No.3600-00). Images were analyzed and presented as before (5).

Antibodies

Primary antibodies were phospho-mTOR (Santa Cruz Biotechnology, Cat.No.101738, 1:1000), mTOR (Pierce, Cat.No.PA534663, 1:1000), ARC (Cedarlane-Synaptic Systems, Cat.No.156003(SY) 1:1000), phospho-AKT (Cell Signaling Technology, Cat.No.9271S, 1:1000), AKT (Cell Signaling Technology, Cat.No.9272S, 1:1000), phospho-ERK1/2 (Cell Signaling Technology, Cat.No.9101S, 1:1000), ERK1/2 (Cell Signaling Technology, Cat.No.9102S, 1:1000), phospho-4EBP1 (Cell Signaling Technology, Cat.No.9459S, 1:1000), 4EBP1 (Cell Signaling Technology, Cat.No.9452S, 1:1000). Actin (Sigma, Cat.No.A2228, 1:20000) and GAPDH (Abcam, Cat.No.ab8245, 1:10000) were used as loading controls. Secondary antibodies were goat anti-rabbit (Pierce, Cat.No.31462, 1:10000) and goat anti-mouse (AnaSpec, Cat.No.28173, 1:10000). All antibodies were diluted in I-Block^{TM} protein-based blocking reagent (Applied Biosystems, Cat.No.T2015).

Blots were reacted with primary antibodies against the following proteins to monitor activation of the translation machinery: 4-EBP1, phospho-4-EBP1, protein kinase B (AKT), phospho-AKT, the mechanistic target of rapamycin (mTOR), phospho-mTOR, extracellular-signal-regulated kinase (ERK)1/2 and phospho-ERK1/2 and against the activity-regulated cytoskeleton-associated protein (ARC) to monitor protein synthesis. All blots were reacted with GAPDH and actin as loading controls. Results from all western blots with phospho-specific antibodies were normalized against the expression levels of the corresponding native proteins, whose expression levels were not changed by the PCP or DHPG treatments. ARC protein levels were normalized against GAPDH or actin, which were used as loading controls. Data were calculated as percent change compared to the control condition from six independent experiments.

Statistical Analyses

Comparisons were performed by Student's t test, one-way, and two-way ANOVAs, as appropriate. When needed, Tukey's and Dunnett's post-hoc comparisons were performed. Statistical significance was accepted when $p < 0.05$.

SUPPLEMENTARY DISCUSSION

Procognitive antipsychotic drug development

The PCP-induced abnormalities in cognitive behavior, neural discoordination, and molecular signaling demonstrate there may be utility of the acute PCP animal model for studying the core cognitive symptoms of schizophrenia, which we understand contrasts with the waning interest in PCP in light of prior lack of success, and the availability of more selective antagonist compounds and genetic models of hypothesized etiological causes of schizophrenia. Indeed, acute PCP intoxication has been the most extensively used experimental preparation for antipsychotic drug development, where the focus has been on treating the positive symptoms of psychosis. Given

the current interest to develop drugs that target cognitive symptoms in schizophrenia, the present demonstration of a set of PCP-induced cognition related deficits indicates that the use of the PCP model can also be extended to the search for a procognitive antipsychotic. In this regard, it is important to consider that PCP-induced cognitive control deficits can be dissociated from the long-established hyperlocomotion that is thought to model the positive symptoms (6). Infusing PCP directly into the hippocampus impaired cognitive control measured by place avoidance without causing hyperlocomotion, so long as the drug increased 60-100 Hz mid-frequency gamma oscillations (6). The dissociation could help understand why treatment strategies that were developed by targeting hyperactivity and other sensorimotor abnormalities turned out to be inadequate for the cognitive symptoms (21). The present work and related work of Kao et al., (2017) also provides compelling evidence that although NMDAR antagonism spares established memory, it can devastate the judicious use of information when sources of cognitive interference abound such as during two-frame avoidance or initial place learning in the water maze (22, 23). These findings suggest it may be necessary to investigate cognitive control, in addition to learning and memory *per se*, in research directed at schizophrenia (24) as well as other fields (4, 25) as predicted by the discoordination hypothesis (26-28). The electrophysiological findings provide unambiguous evidence that PCP discoordinates electrical neural activity between neurons (Fig. 1,2) with minimal consequences on the response characteristics of individual cells during normal behavior (6) as predicted by the discoordination hypothesis. The findings with anisomycin and MPEP, as well as our direct assays of the translation molecular machinery, indicate that the PCP-induced cognitive impairment is mediated or at least modulated by dysregulated translation, consistent with findings in DISC1 and PKR-like ER kinase (PERK) mutant mice that mimic gene alterations in schizophrenia and implicate excessive translation in the cognitive deficits associated with the disease (29, 30). Despite stimulating translation and consequent BDNF synthesis, which has antidepressant effects when elicited by ketamine, another uncompetitive NMDAR-antagonist (31, 32), the effects of PCP are debilitating. This highlights the need for caution and rigor in

attempts to develop mechanistically-related ketamine antidepressant therapies. The findings that doses of PCP that impair cognition also discoordinate neural activity suggest that normalizing neural discoordination could be a procognitive therapeutic target. Although this may be accomplished by correcting a proximal cause such as dysregulated translation, procognitive effects can also be achieved by correcting the discoordination itself, using pharmacological treatment to restore excitation-inhibition balance (33), or even by harnessing the plasticity that can accompany appropriate cognitive experience (25, 34, 35).

SUPPLEMENTARY REFERENCES

1. Chung A, Dahan N, Alarcon JM, Fenton AA (2017): Effects of regulatory BC1 RNA deletion on synaptic plasticity, learning, and memory. *Learn Mem*. 24:646-649.
2. Olypher AV, Klement D, Fenton AA (2006): Cognitive disorganization in hippocampus: a physiological model of the disorganization in psychosis. *J Neurosci*. 26:158-168.
3. Dvorak D, Chung A, Park EH, Fenton AA (2021): Dentate spikes and external control of hippocampal function. *Cell Rep*. 36:109497.
4. Talbot ZN, Sparks FT, Dvorak D, Curran BM, Alarcon JM, Fenton AA (2018): Normal CA1 Place Fields but Discoordinated Network Discharge in a Fmr1-Null Mouse Model of Fragile X Syndrome. *Neuron*. 97:684-697.
5. Jourdi H, Hsu YT, Zhou M, Qin Q, Bi X, Baudry M (2009): Positive AMPA receptor modulation rapidly stimulates BDNF release and increases dendritic mRNA translation. *J Neurosci*. 29:8688-8697.
6. Kao HY, Dvorak D, Park E, Kenney J, Kelemen E, Fenton AA (2017): Phencyclidine discoordinates hippocampal network activity but not place fields. *J Neurosci*. 37:12031–12049.
7. Neymotin SA, Lytton WW, Olypher AV, Fenton AA (2011): Measuring the Quality of Neuronal Identification in Ensemble Recordings. *J Neurosci*. 31:16398-16409.
8. Pachitariu M, Steinmetz N, Kadir S, Carandini M, Harris KD (2016): Kilosort: realtime spike-sorting for extracellular electrophysiology with hundreds of channels. *bioRxiv 061481 (2016)*.
9. Fenton AA, Kao H-Y, Neymotin SA, Olypher AV, Vayntrub Y, Lytton WW, et al. (2008): Unmasking the CA1 ensemble place code by exposures to small and large environments: more place cells and multiple, irregularly-arranged, and expanded place fields in the larger space. *J Neurosci*. 28:11250-11262.
10. Kelemen E, Fenton AA (2010): Dynamic grouping of hippocampal neural activity during cognitive control of two spatial frames. *PLoS Biol*. 8:e1000403
.
11. Ranck JB, Jr. (1973): Studies on single neurons in dorsal hippocampal formation and septum in unrestrained rats. I. Behavioral correlates and firing repertoires. *Exp Neurol*. 41:461-531.
12. Fox SE, Ranck JB, Jr. (1975): Localization and anatomical identification of theta and complex spike cells in dorsal hippocampal formation of rats. *Exp Neurol*. 49:299-313.
13. Press WH, Flannery BP, Teukolsky SA, Vetterling WT (1993): *Numerical Recipes in C: The Art of Scientific Computing*. 2nd ed. Cambridge: Cambridge University Press.

14. Chung S, Lee DD, Sompolinsky H (2018): Classification and Geometry of General Perceptual Manifolds. *Physical Review X*. 8:031003.
15. Pavlowsky A, Alarcon J (2012): Interaction between long-term potentiation and depression in CA1 synapses: temporal constrains, functional compartmentalization and protein synthesis. *PLoS ONE*. 7:e29865.
16. Lesburgueres E, Sparks FT, O'Reilly KC, Fenton AA (2016): Active place avoidance is no more stressful than unreinforced exploration of a familiar environment. *Hippocampus*. 26:1481-1485.
17. Patil ST, Zhang L, Martenyi F, Lowe SL, Jackson KA, Andreev BV, et al. (2007): Activation of mGlu2/3 receptors as a new approach to treat schizophrenia: a randomized Phase 2 clinical trial. *Nat Med*. 13:1102-1107.
18. Topisirovic I, Sonenberg N (2011): mRNA translation and energy metabolism in cancer: the role of the MAPK and mTORC1 pathways. *Cold Spring Harb Symp Quant Biol*. 76:355-367.
19. Lyford GL, Yamagata K, Kaufmann WE, Barnes CA, Sanders LK, Copeland NG, et al. (1995): Arc, a growth factor and activity-regulated gene, encodes a novel cytoskeleton-associated protein that is enriched in neuronal dendrites. *Neuron*. 14:433-445.
20. Zhou M, Baudry M (2006): Developmental changes in NMDA neurotoxicity reflect developmental changes in subunit composition of NMDA receptors. *J Neurosci*. 26:2956-2963.
21. Carter CS, Barch DM (2007): Cognitive neuroscience-based approaches to measuring and improving treatment effects on cognition in schizophrenia: the CNTRICS initiative. *Schizophr Bull*. 33:1131-1137.
22. Bannerman DM, Good MA, Butcher SP, Ramsay M, Morris RG (1995): Distinct components of spatial learning revealed by prior training and NMDA receptor blockade. *Nature*. 378:182-186.
23. Saucier D, Cain DP (1995): Spatial learning without NMDA receptor-dependent long-term potentiation. *Nature*. 378:186-189.
24. Gilmour G, Arguello A, Bari A, Brown VJ, Carter C, Floresco SB, et al. (2013): Measuring the construct of executive control in schizophrenia: defining and validating translational animal paradigms for discovery research. *Neurosci Biobehav Rev*. 37:2125-2140.
25. Chung A, Jou C, Grau-Perales A, Levy ERJ, Dvorak D, Hussain N, et al. (2021): Cognitive control persistently enhances hippocampal information processing. *Nature*. 600:484-488.
26. Fenton AA (2015): Excitation-inhibition discoordination in rodent models of mental disorders. *Biol Psychiatry*. 77:1079-1088.
27. Phillips WA, Silverstein SM (2003): Convergence of biological and psychological perspectives on cognitive coordination in schizophrenia. *Behav Brain Sci*. 26:65-82; discussion 82-137.
28. Fenton AA (2008): Neural coordination and psychotic disorganization In: Holscher C, Munk MH, editors. *Information Processing by Neuronal Populations*. London: Cambridge University Press, pp 387-408.
29. Zhou M, Li W, Huang S, Song J, Kim JY, Tian X, et al. (2013): mTOR Inhibition ameliorates cognitive and affective deficits caused by Disc1 knockdown in adult-born dentate granule neurons. *Neuron*. 77:647-654.
30. Trinh MA, Kaphzan H, Wek RC, Pierre P, Cavener DR, Klann E (2012): Brain-specific disruption of the eIF2alpha kinase PERK decreases ATF4 expression and impairs behavioral flexibility. *Cell Rep*. 1:676-688.
31. Takahashi M, Kakita A, Futamura T, Watanabe Y, Mizuno M, Sakimura K, et al. (2006): Sustained brain-derived neurotrophic factor up-regulation and sensorimotor gating abnormality induced by postnatal exposure to phencyclidine: comparison with adult treatment. *J Neurochem*. 99:770-780.
32. Autry AE, Adachi M, Nosyreva E, Na ES, Los MF, Cheng PF, et al. (2011): NMDA receptor blockade at rest triggers rapid behavioural antidepressant responses. *Nature*. 475:91-95.

33. Lee H, Dvorak D, Kao HY, Duffy AM, Scharfman HE, Fenton AA (2012): Early cognitive experience prevents adult deficits in a neurodevelopmental schizophrenia model. *Neuron*. 75:714-724.
34. Lee H, Dvorak D, Fenton AA (2014): Targeting Neural Synchrony Deficits is Sufficient to Improve Cognition in a Schizophrenia-Related Neurodevelopmental Model. *Front Psychiatry*. 5:15.
35. Pavlowsky A, Wallace E, Fenton AA, Alarcon JM (2017): Persistent modifications of hippocampal synaptic function during remote spatial memory. *Neurobiol Learn Mem*. 138:182-197.
